# Supplementary material for: Nitric Oxide Trickle Drives Heme into Hemoglobin and Muscle Myoglobin
Source: Cells. 2022 Sep 12;11(18):2838. doi: 10.3390/cells11182838 (PMC9496899; doi:10.3390/cells11182838)
Supplement: Supplementary file 1 [file cells-11-02838-s001.zip › cells-1917775-supplementary.pdf]

Supplementary Information: Figures S1-S5

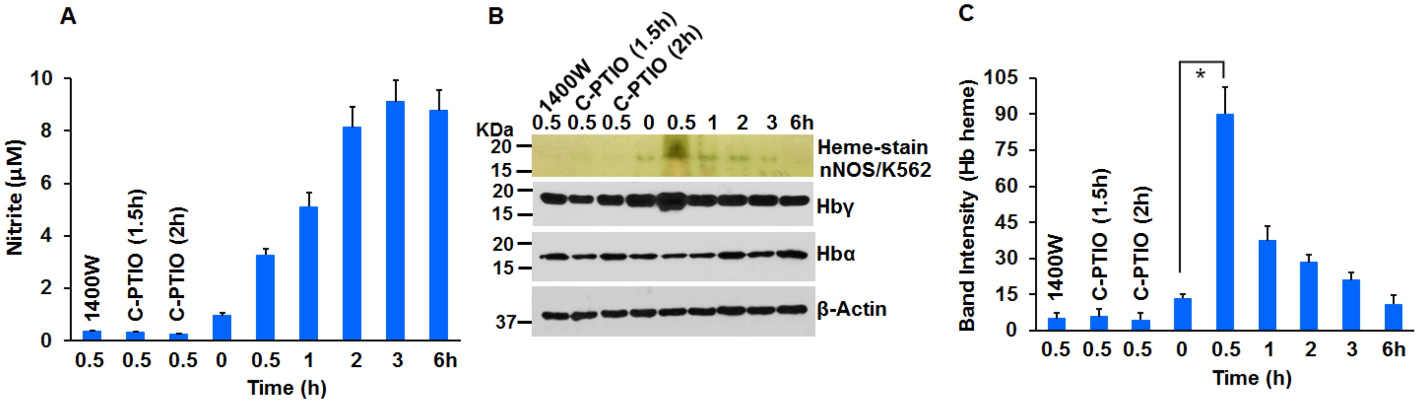

Figure S1. NO induces heme-insertion into fetal Hb and this heme-insertion is inhibited in the presence of NO scavenger, Carboxy-PTIO. Stable lines of HEK cells expressing nNOS were cultured and activated with Ca ionophore for 30 min, -/+ NO scavenger, Carboxy-PTIO or -/+ NOS inhibitor (1400W) before being co-cultured with K562 cells in a transwell for various lengths of time between 0-6 h. The cultures were then harvested and generated supernatants were assayed for protein expression by westerns, Hb heme by heme-stain and nNOS generated NO (as nitrite) by an ozone based chemiluminescent assay. Panel (A) NO estimation as nitrite by a chemiluminescent assay. Panel (B) Protein expression of Hbα/γ, loading control β-actin and Hb heme-stains as indicated. Panel (C) Mean densitometries of heme-stains from three independent experiments. Values depicted are mean n=3, ± SD. *$p < 0.05$, by one-way ANOVA. Molecular weight markers (KDa) are depicted at the left of gel bands throughout the figure legends.

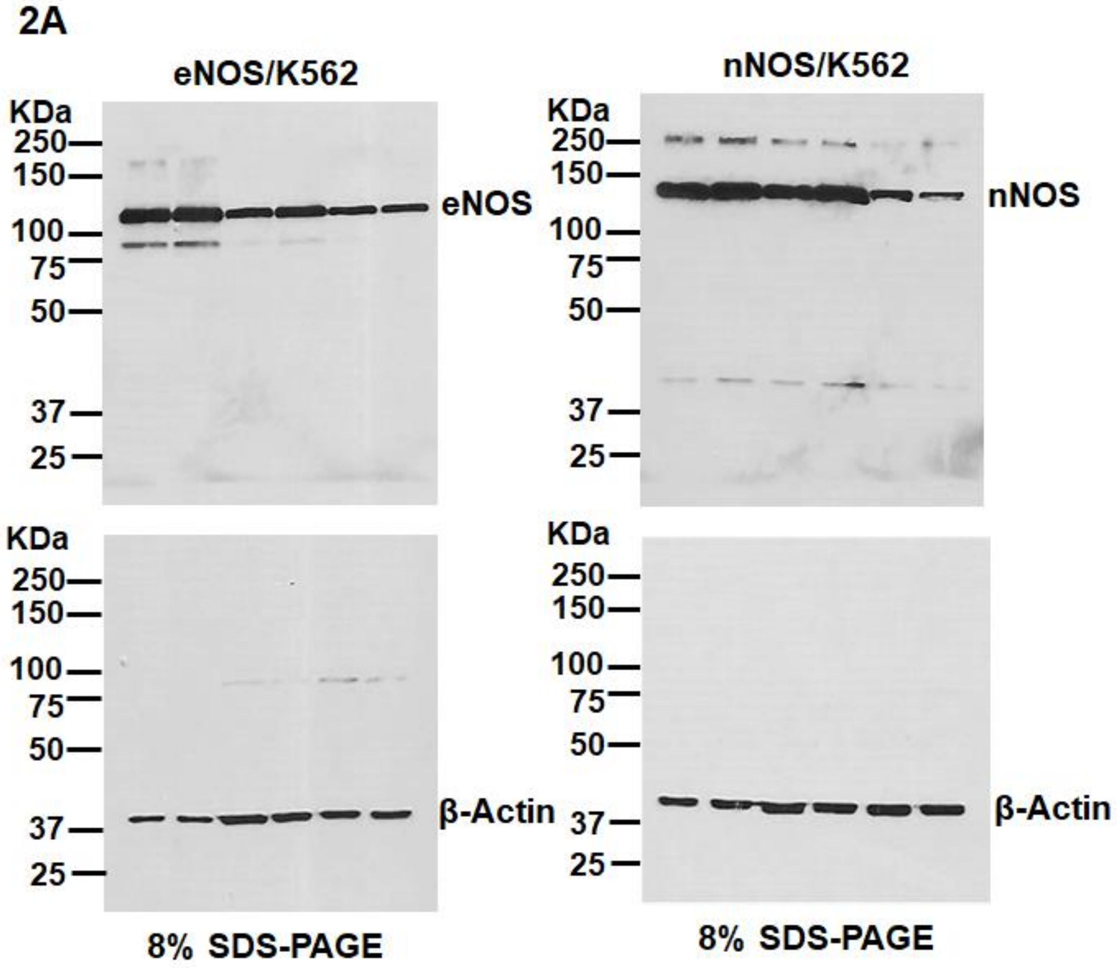

Figure S2. Full gels of western blots as depicted in figure 2A. Molecular weight markers (KDa) are depicted at the left of the gels.

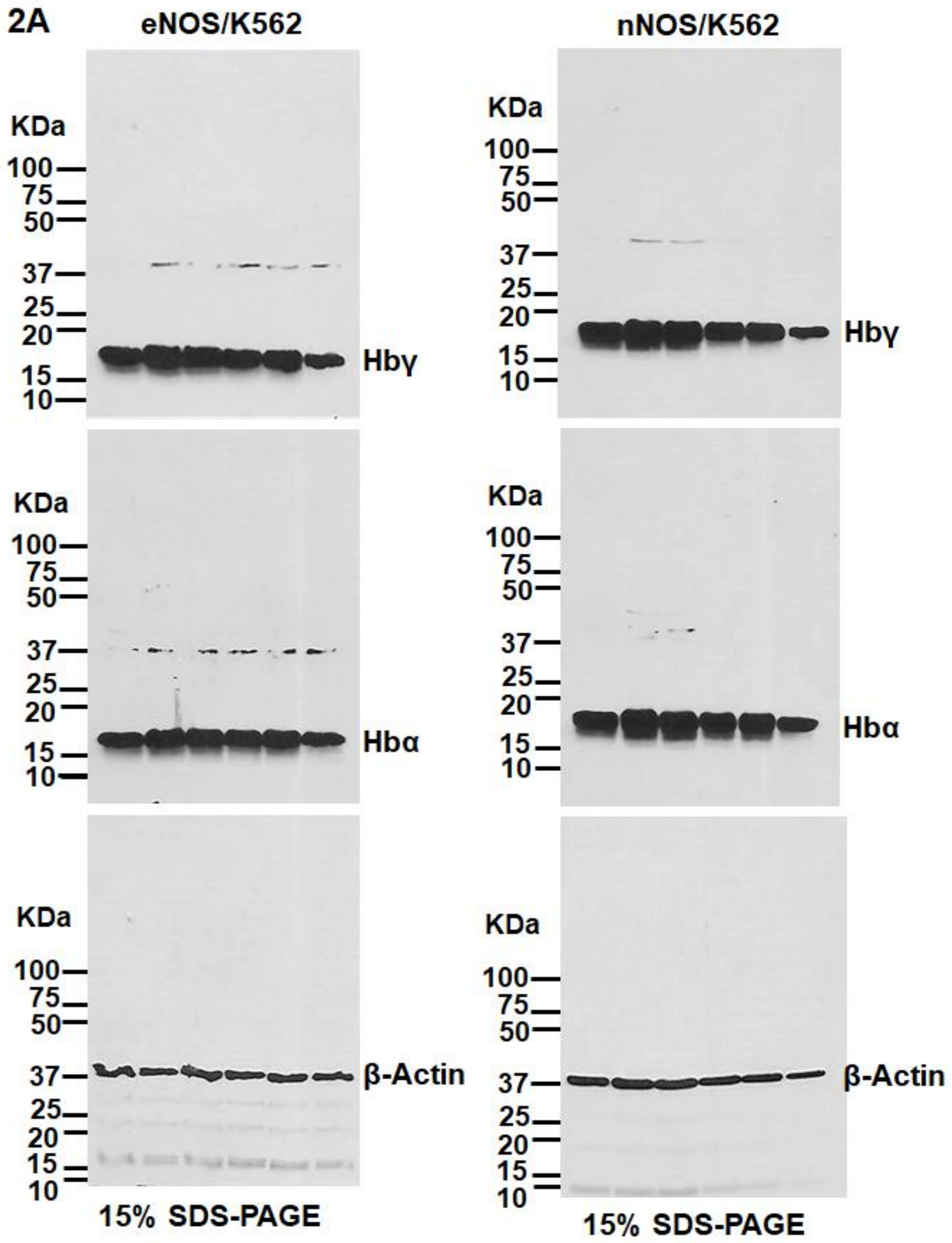

Figure S3. Full gels of western blots as depicted in figure 2A.

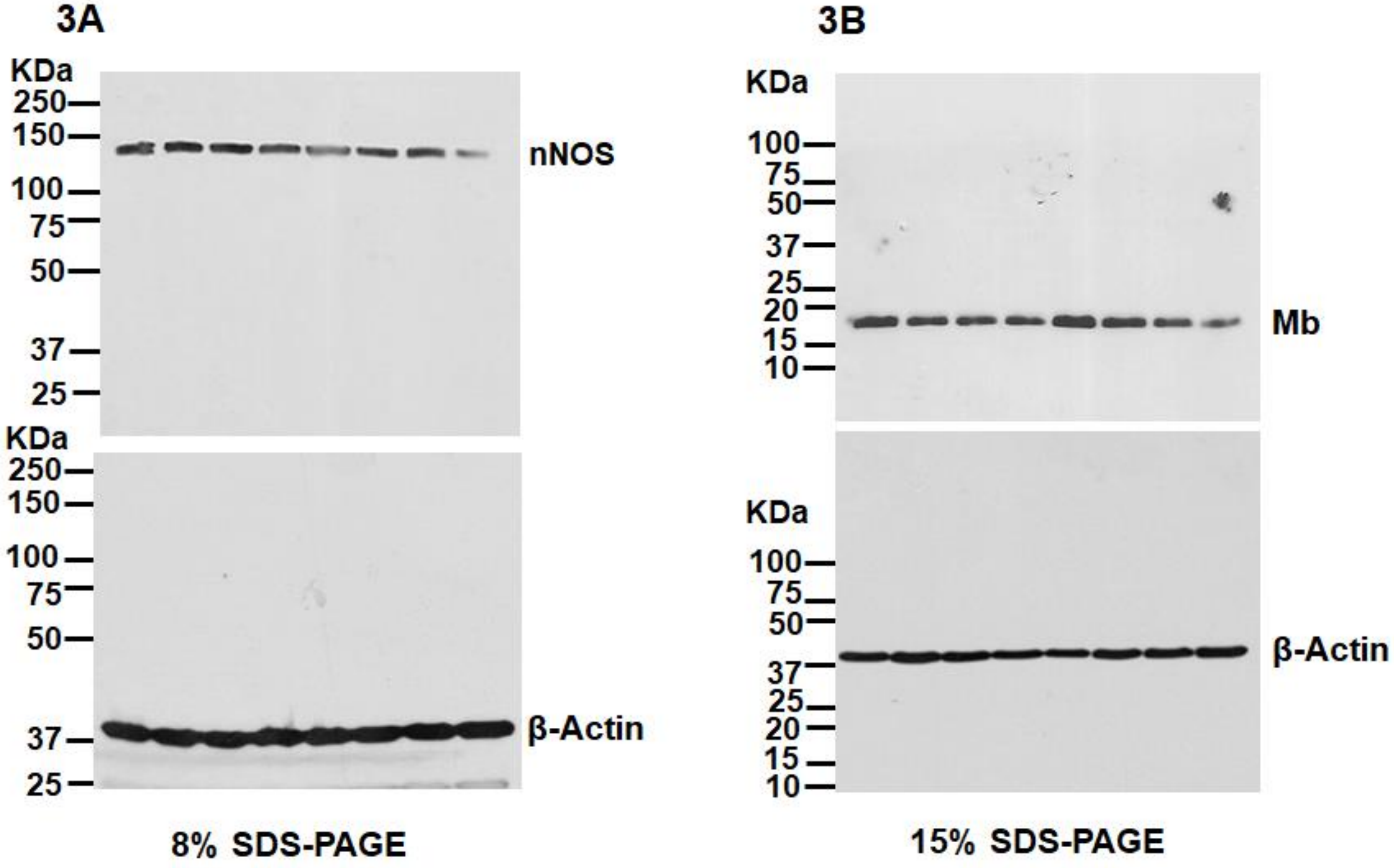

Figure S4. Full gels of western blots as depicted in figures 3A and B.

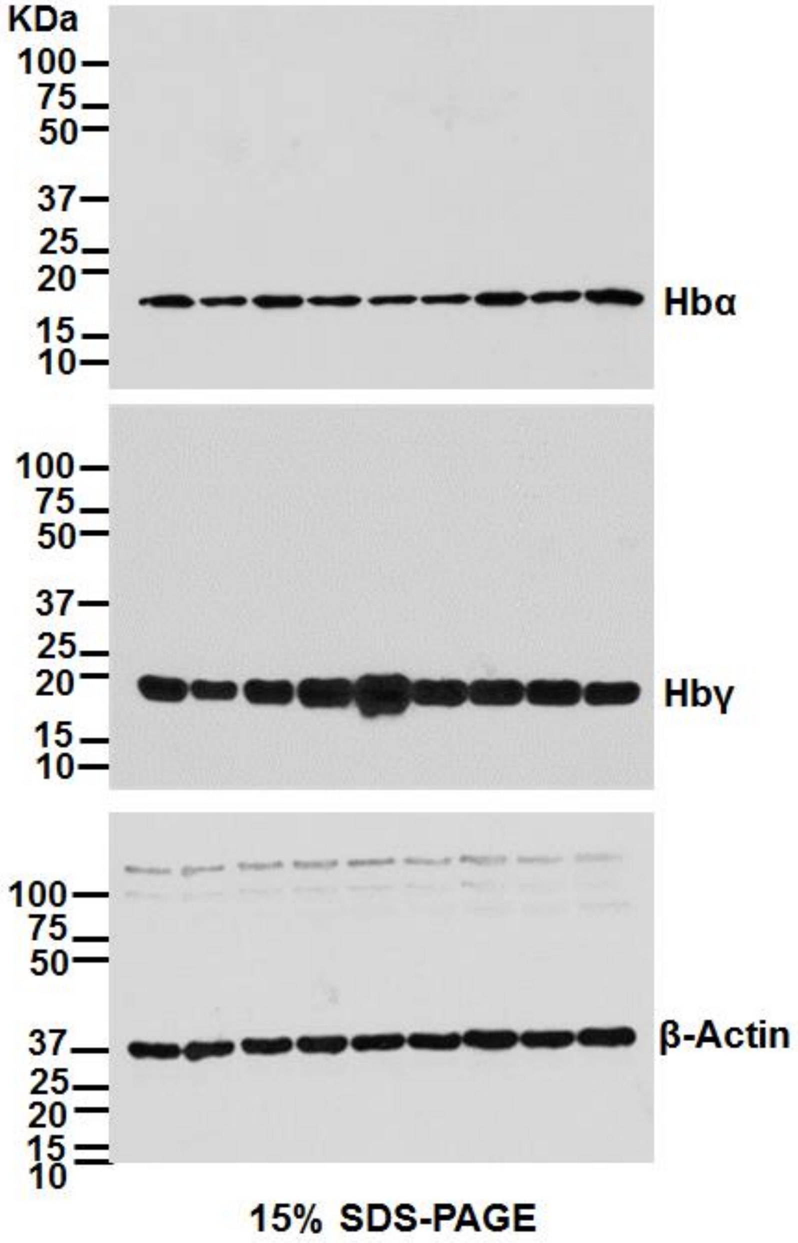

Figure S5. Full gels of western blots as depicted in figure S1B.
